# Honeybees' Speed Depends on Dorsal as Well as Lateral, Ventral and Frontal Optic Flows

**DOI:** 10.1371/journal.pone.0019486

**Published:** 2011-05-12

**Authors:** Geoffrey Portelli, Franck Ruffier, Frédéric L. Roubieu, Nicolas Franceschini

**Affiliations:** Biorobotic Department, Institute of Movement Science, CNRS/Aix-Marseille II University, Marseille, France; Imperial College London, United Kingdom

## Abstract

Flying insects use the optic flow to navigate safely in unfamiliar environments, especially by adjusting their speed and their clearance from surrounding objects. It has not yet been established, however, which specific parts of the optical flow field insects use to control their speed. With a view to answering this question, freely flying honeybees were trained to fly along a specially designed tunnel including two successive tapering parts: the first part was tapered in the vertical plane and the second one, in the horizontal plane. The honeybees were found to adjust their speed on the basis of the optic flow they perceived not only in the lateral and ventral parts of their visual field, but also in the dorsal part. More specifically, the honeybees' speed varied monotonically, depending on the minimum cross-section of the tunnel, regardless of whether the narrowing occurred in the horizontal or vertical plane. The honeybees' speed decreased or increased whenever the minimum cross-section decreased or increased. In other words, the larger sum of the two opposite optic flows in the horizontal and vertical planes was kept practically constant thanks to the speed control performed by the honeybees upon encountering a narrowing of the tunnel. The previously described ALIS (“AutopiLot using an Insect-based vision System”) model nicely matches the present behavioral findings. The ALIS model is based on a feedback control scheme that explains how honeybees may keep their speed proportional to the minimum local cross-section of a tunnel, based solely on optic flow processing, without any need for speedometers or rangefinders. The present behavioral findings suggest how flying insects may succeed in adjusting their speed in their complex foraging environments, while at the same time adjusting their distance not only from lateral and ventral objects but also from those located in their dorsal visual field.

## Introduction

There exists strong evidence that flying insects perceive and use the optic flow to control their flight [1]–[8]. The optic flow is the *angular velocity* at which any environmental feature sweeps past the insect's eyes as the result of its own motion [1], [3], [6], [9], [10]. The *translational* optic flow perceived in a given direction depends on the ratio between the relative speed and the distance to the environment in that direction [11]. This sensitivity to the translational optic flow enables insects to navigate safely and efficiently in unfamiliar environments. Insects' terrain following and landing abilities have been explained in terms of holding the *ventral optic flow* constant by consistently adjusting the *lift* [12]. It has also been established that honeybees flying along a corridor keep a safe clearance from the walls [6], [13], [14] and from the ground [15]. However, although many studies have focused on this topic, it is not yet clear how insects manage to adjust their *speed* based on the visually perceived optic flow [4], [16], [17], [18], [19]. Honeybees trained to fly along a tapered tunnel were found to reduce their speed when the tunnel narrowed and to accelerate when the tunnel widened [16]. The authors of the latter study concluded that “*honeybees strive to hold the angular velocity of the image in the lateral region of the eyes constant*” [16]. When flying through a tunnel equipped with moving walls, honeybees have also been found to adjust their speed “*so as to hold constant the image angular velocity in the eye*” [17], [20]. Other evidence suggests that the *ventral* optic flow also contributes significantly to the speed control process [16], [20], [21]. The latter authors used various tunnels, the floor of which was lined with stationary patterns of various kinds, such as 2-D patterns providing abundant ventral optic flow cues, axial patterns providing only a few ventral optic flow cues and a homogeneous pattern providing hardly any optic flow cues. Honeybees were found to fly at a lower height and a higher speed on average when few ventral optic flow cues were available.

**Figure 1:**
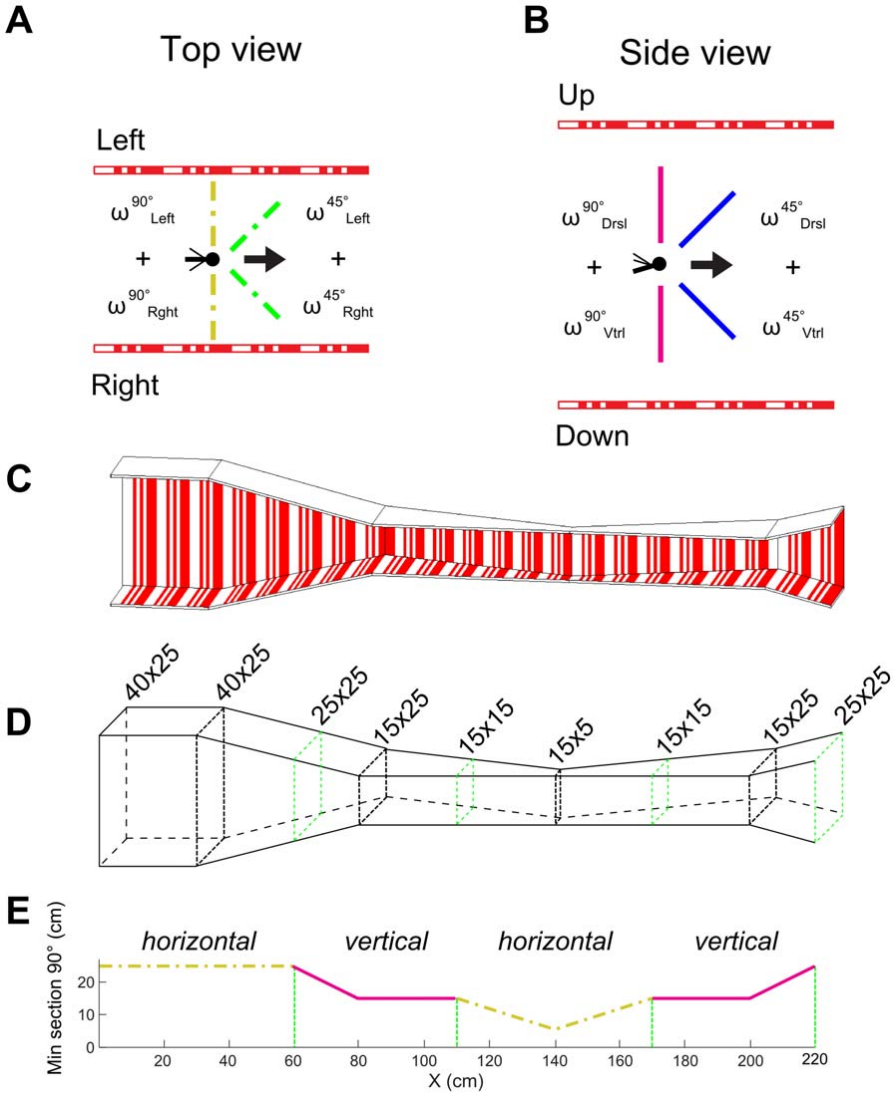
Experimental flight tunnel. (A) Top view of the tunnel. The honeybee flies into the tunnel. The left optic flow ω*^90°^*_Left_ and the right optic flow ω*^90°^*_Rght_ are generated by the contrasts on the side walls. The sum of these opposite optic flows at 90° is Σω*^90°^*_Lat_ (dash-dotted yellow line). The left optical flow ω*^45°^*_Left_ and the right optical flow ω*^45°^*_Rght_ are generated at an angle of 45° with respect to the tunnel x-axis. Their sum is Σω*^45°^*_Lat_ (dash-dotted green line). (B) Side view of the tunnel. The honeybee flies into the tunnel. The dorsal optic flow ω*^90°^*_Drsl_ and the ventral optical flow ω*^90°^*_Vtrl_ are generated by the contrasting stripes on the ceiling and the floor of the tunnel, respectively. The sum of these optic flows at an angle of 90° is Σω*^90°^*_Vert_ (magenta line). The dorsal optic flow ω*^45°^*_Drsl_ and the ventral optical flow ω*^45°^*_Vtrl_ are generated at an angle of 45° with respect to the tunnel x-axis. Their sum is Σω*^45°^*_Vert_ (blue line). (C–D) Perspective view of the whole doubly-tapered tunnel. Two tapered zones occur in this tunnel: the first one is tapered in the vertical plane (from 30 cm to 80 cm, tapering angle 14°), and the second, in the horizontal plane (from 80 cm to 200 cm, tapering angle 18°). (E) Minimum section of the tapered tunnel along the abscissa. Because of the way this particular tunnel was designed, the minimum section was encountered alternately in the horizontal plane (dash-dotted yellow line) and the vertical plane (magenta line).

Based on these studies, one might expect the *lateral* optic flow to affect honeybees' flight speed and the *ventral* optic flow to affect both their flight speed and their flight height. In order to combine all these findings in a single control model, we recently developed the ALIS autopilot [22] (ALIS stands for “AutopiLot using an Insect based vision System”), which is based on the concept of *optic flow regulation* [23]. The *optic flow regulator* is a feedback control system that strives to maintain the perceived optic flow at a constant reference value: the optic flow set point. The ALIS control scheme actually incorporates two optic flow regulators: the first one controls the vertical and horizontal positions, while the second one controls the speed. The first optic flow regulator relies on the largest optic flow (left, right, dorsal, or ventral), and the second one relies on the *larger of the two sums of opposite optic flows* (i.e., “left + right” optic flows or “ventral + dorsal” optic flows). Consequently, it is the plane (horizontal or vertical) affording the larger of the two optic flow sums that will constrain the bee's speed. To test the relevance of the ALIS model, we designed a doubly-tapered flight tunnel comprising two successive tapering parts that freely flying honeybees would encounter: in the first part, a gradual constriction occurred in the vertical plane, and in the second one, a gradual constriction occurred in the horizontal plane (see figure 1C–1D, a photograph of a honeybee flying along the doubly-tapered tunnel in Figure S1, as well as an animated 3D view of the doubly-tapered tunnel in Movie S1). The ALIS model predicts that a honeybee flying along either of these two tapered sections will adjust its speed at all times on the basis of the minimum local cross-section of the tunnel, whether the latter occurs in the vertical or horizontal plane.

In the experiments carried out here, freely flying honeybees were trained to fly along the doubly-tapered tunnel. Their trajectories were recorded and special attention was paid to how the honeybees adjusted their speed as they crossed the various sections of the tunnel. Lastly, the flight performances of a bee were simulated in the same doubly-tapered tunnel on the basis of our ALIS model, and the actual and the simulated flight profiles were compared.

## Materials and Methods

### Doubly tapered flight tunnel

The floor, roof and left wall of the outdoor flight tunnel used in this study consisted mainly of planks lined with red and white stripes. The right wall consisted of thin white insect netting lined with stripes consisting of a red Gelatin filter (Lee Filters HT019), through which the honeybee's flight paths could be seen and video-recorded. The flight tunnel was 220 cm long, 40 cm high and 25 cm wide at the entrance.

**Figure 2:**
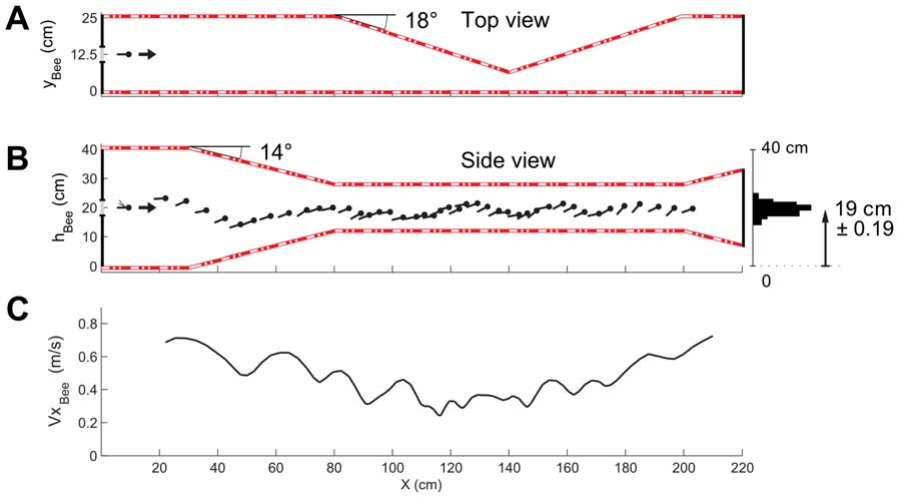
Typical trajectory of an individual honeybee in the doubly-tapered tunnel. (A) Top view of the tunnel showing the entrance of the honeybee, and the tapering in the horizontal plane at a distance of 80 cm to 200 cm from the entrance. (B) Side view of the actual trajectory of a honeybee, plotted every 100 ms. The honeybee's course was fairly well centered in the tunnel (mean height *h* = 19±0.19 cm). (C) Honeybee's speed as a function of the distance along the abscissa *x*. The honeybee decreased its speed as the tunnel narrowed, regardless of whether the narrowing was in the vertical or the horizontal plane. The honeybee then increased its speed as the tunnel widened.

The tunnel comprised two successive tapering parts (Figure 1C–1D). In the first of these parts, the narrowing occurred in the vertical plane with a 14° tapering angle (Figure 2B) and involved both the roof and the floor. It started 30 cm from the entrance and the maximum constriction (15 cm high by 25 cm wide) occurred 80 cm from the entrance. In the second tapering part, the narrowing occurred in the horizontal plane with a tapering angle of 18° (Figure 2A) and involved only the left wall (the right wall made of insect netting remained straight). This part started 80 cm from the entrance and the maximum constriction (15 cm high by 5 cm wide) was reached in this case 140 cm from the entrance. Beyond the second constriction, the tunnel widened out horizontally until reaching a section 15 cm high by 25 cm wide at a distance of 200 cm from the entrance. From 200 cm to 220 cm, the tunnel then widened vertically until reaching a section 25 cm high by 25 cm wide. The diagram in figure 1E shows that the minimum section was first the horizontal section (dash-dotted yellow line), then the vertical section (continuous magenta line), then the horizontal section again (dash-dotted yellow line) and lastly, the vertical section (continuous magenta line).

Two manually operated openings (5×5 cm) centered at mid height and mid width gave single honeybees entry to the tunnel and access to the reward, respectively (only the entrance opening is shown in figures 2A,2B). This outdoor flight tunnel was oriented to the north and received only indirect illumination (and no direct sunlight). A photograph of a honeybee flying along the doubly-tapered tunnel is presented in Figure S1; an animated 3D view shows the overall geometry of the doubly-tapered tunnel in Movie S1.

### Pattern

The pattern on the walls of the tunnel consisted of red and white stripes oriented perpendicularly to the flight direction. Since honeybees are devoid of red-sensitive photoreceptors [24], they perceive red stripes as gray shades. These red stripes had two different widths (1 cm and 3 cm), forming a uniform 10 cm-wide pattern that was repeated periodically, as shown in Figure 1. The angle subtended by the stripes ranged from 5.7° to 53° (a 1–10 cm pattern viewed from a distance of 10 cm, respectively) and from 1.4° to 14.2° (a 1–10 cm pattern viewed from a distance of 40 cm, respectively). The Michelson contrast between the red and white stripes was *m* = 0.47 on the planks and *m* = 0.25 on the insect netting. Contrast was measured using a photodiode equipped with a green band-pass filter (Kodak Wratten N°61), the transmission spectrum of which closely matched the spectral sensitivity of the honeybee's green photoreceptors [24], which are the receptors involved in motion vision [25], [26], [27], [28]. A red filter placed in front of the camcorder monitoring the honeybees' trajectories through the insect netting was used to optimize the contrast between the honeybee and the background.

### Experimental procedure

Groups of four to six freely flying honeybees (*Apis mellifera*) were color-marked and trained outdoors to enter the tunnel and fly along it to collect sugar solution at the opposite end (see Figure S1). Once honeybees had received about 30 rewards, their flight path was recorded with the digital camera from the insect-netting side, on their way to the reward. Only one honeybee at a time was allowed to enter the tunnel during each recording session. The camcorder was triggered at the moment the honeybee entered the tunnel. During the recordings, the white door giving access to the reward remained seamlessly closed to rule out the presence of any uncontrolled attractive cues.

### Video recordings and flight path analysis

The honeybees' trajectories were filmed at a rate of 20 frames per second (Ts = 50 ms) with a high-resolution digital black-and-white CMOS camera (Prosilica EC1280, 1/3” sensor size) equipped with a Fujinon HF12.5HA-1B lens. The camera was placed sideways, 265 cm from the insect netting. The small field of view (21°44′×16°23′) covered the whole height of the tunnel, from abscissa *x* = 20 cm to abscissa *x* = 210 cm. The lens had a maximum barrel distortion of 1.48% along x on the extreme upper border of the field of view and a maximum barrel distortion of 0.8%, vertically, on the extreme right and left border of the field of view. However, the trajectories were recorded in the middle of the field of view, where the maximum lens distortion was only 0.23% along x and 0.8%, vertically. The effect of perspective foreshortening was therefore neglected. Image sequences were processed and analyzed using a custom-made Matlab program. In any sequence of images, this program automatically determines the honeybees' flight height (*h*) in each frame as a function of the abscissa (*x*) along the tunnel axis, thus allowing the honeybee's trajectory in the vertical plane to be reconstructed. The honeybees' instantaneous ground speed (*Vx*_Bee_) was computed on each abscissa *x* using a four-point derivative smoothing filter (*Vx*_Bee_(*t*) = (2*x*_Bee_(*t*−2)+*x*_Bee_(*t*−1)−*x*_Bee_(*t*+1)−2*x*_Bee_(*t*+2))/10Ts), as was the honeybees' instantaneous vertical speed (*Vh*_Bee_(*t*) = (2*h*_Bee_(*t*−2)+*h*_Bee_(*t*−1)−*h*_Bee_(*t*+1)−2*h*_Bee_(*t*+2))/10Ts).

### Analysis

The honeybees were assumed to fly taking a laterally centered course, aligned with the tunnel's x-axis, as found to occur in similar (narrow) tunnels [6], [13], [16], [19]. Their head orientation was also assumed to remain practically fixed and aligned with the tunnel axis. This assumption is supported by findings obtained on another hymenopteran [29] and on Dipterans [30]–[34], showing that insects produce consistent head counter-rotations that compensate for their body's yaw, pitch and roll motions, and thereby stabilize their gaze relative to the environment. These gaze locking properties have been observed in many species [29].

The parameters used in the present analysis were the honeybees' flight height (*h*) and their flight speed (*V*_Bee_). The latter was resolved into the ground speed *Vx*_Bee_ and the vertical speed *Vh*_Bee_. Depending on the honeybees' position (*x*, *h*), their distances from the four walls of the tunnel were determined at a viewing angle of 90° (*D^90°^_Lft_* = distance from the left wall, *D^90°^_Rght_* = distance from the right wall, *D^90°^_Drsl_* = distance from the roof, and *D^90°^_Vtrl_* = distance from the floor). The translational optic flows perceived at viewing angles of 90° can be defined as the speed-to-distance ratio according to the following equation: *ω^90°^_i_* = *Vx*_Bee_/*D^90°^_i_*, where *i* ∈{*Rght*, *Lft*, *Drsl*, *Vtrl*}, taking the distances from the walls at an angle of 90° and *Vx*_Bee_ the bee's ground speed (Figure 1A–1B).

Experiments by Srinivasan et al. [13] have provided evidence that honeybees flying along a tunnel monitor the optic flow chiefly via the lateral parts of their visual field. However, the honeybee's panoramic compound eye is able to perceive the environment in many other directions, which provides the bee with relevant optic flow information to control their speed, as recently shown for the frontal visual field by Baird et al (2010) [19]. Studies on recently designed insect-inspired aerial robots based on optic flow sensing mechanisms showed that the optic flow perceived at 45° from the heading direction is a particularly relevant and reliable parameter for controlling the course of a micro aircraft [35]. It can also be used for anticipation purposes and to improve the efficiency in terms of obstacle avoidance [36]. We therefore investigated the possible role of the optic flow perceived by honeybees at an angle of 45°, either laterally, ventrally or dorsally in the context of honeybees' speed control. The translational optic flows generated at 45° in the honeybees' frontal field of view can be defined according to the following equation: *ω^45°^_i_* = (*V*_Bee_ .sinψ_i_)/*D^45°^_i_* with *i* ∈ {*Rght*, *Lft*, *Drsl*, *Vtrl*}, where *V*_Bee_ is the honeybee's speed, resolved into the ground speed *Vx_Bee_* and vertical speed *Vh_Bee_*, and ψ_i_ is the angle between the honeybee's speed vector and the gaze direction under consideration (ψ_i_ = 45° + atan(*Vh_Bee_*/*Vx_Bee_*) for *i* ∈ {*Drsl*, *Vtrl*} and ψ_i_ = 45° for *i *∈ {*Rght*, *Lft*} as *Vy_Bee_* is unknown), and *D^45^_i_* are the distances between the bee and the four surfaces at an angle of 45°, as shown in Figure 1A–1B.

**Figure 3:**
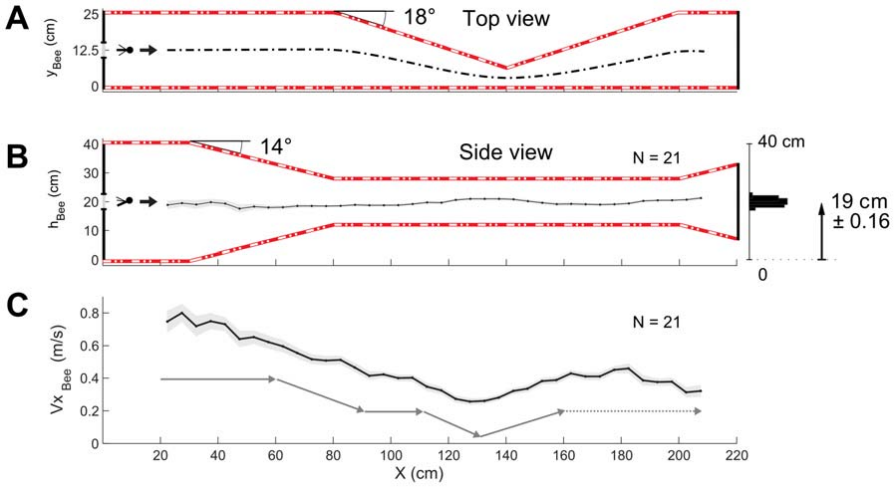
Mean vertical trajectory and mean speed of the 21 honeybees in the doubly-tapered tunnel. (A) Top view of the tunnel showing the entrance of the honeybee, the part tapering in the horizontal plane (from 80 cm to 200 cm) and the assumed trajectory of the insect in the horizontal plane (see text). (B) Side view of the tapered tunnel, showing in particular the vertical constriction. The mean flight path of the honeybees is plotted as a function of the distance along the abscissa. The insects' mean trajectory can be seen to be practically vertically centered throughout the tunnel (mean height *h* = 19±0.16 cm). (C) Ground speed profile along the tunnel. The honeybees decreased their speed as the tunnel narrowed and increased their speed as it widened. The faded trace around the curves gives ± the standard error of the mean (s.e.m.). The gray profile underneath the main curve shows the overall flight speed pattern as shown by the analysis.

In Figure 3B–3C, the flight height *h* and the ground speed *Vx*_Bee_ are each plotted as a function of the abscissa *x*. Height and speed were averaged at 5-cm intervals along a distance of 190 cm: each of the 38 data points plotted is the mean value of the honeybee's individual height and speed values, respectively. A one-way repeated-measures ANOVA was performed on the 38 *mean height* data points and the 38 *mean speed* data points versus the position *x* in the tunnel. To further investigate the differences between points, a TukeyHSD post-hoc test was applied. In these analyses, significance level was taken to be α = 0.05. The faded colors around the curves give ± the standard error of the mean (s.e.m.).

**Figure 4:**
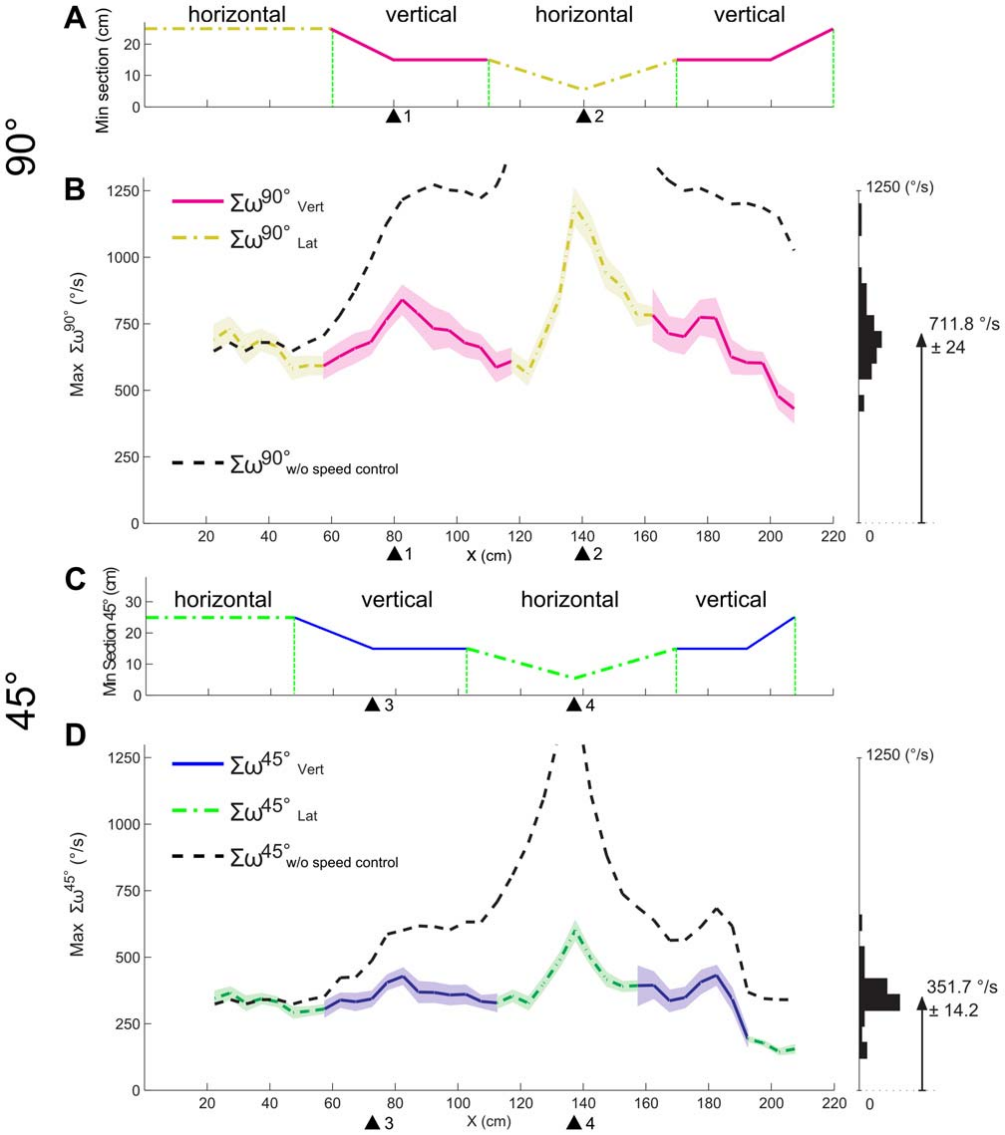
Analysis of the *larger of the two optic flow sums* perceived by the 21 honeybees at an angle of 90° and at 45° with respect to the tunnel x-axis, in comparison to the minimum section of the tunnel at each point along the tunnel. (A) Minimum section at 90° of the tapered tunnel as a function of the distance along the abscissa. The minimum section at 90° was alternately in the horizontal plane and vertical plane. (B) Larger of the two optic flow sums in the *horizontal* plane (dash-dotted yellow line) and the *vertical* plane (magenta line), (mean(Max Σ*ω^90°^*) = 711.8±24°/s, the highest value peaks at Max(Max Σ*ω^90°^*) = 1192°/s), as well as the larger optic flow that would have been experienced theoretically at 90° at a constant ground speed (0.74 m/s), i.e., without the use of any speed control system by the bee (dashed black line, mean(MaxΣ*ω^90°^_w/o_*_SpeedControl_) = 1258.2±85°/s, the highest value peaks at Max(Max Σ*ω^90°^_w/o_*_SpeedControl_) = 2971°/s). (C) Minimum section profile of the tapered tunnel, encountered at an angle of 45° from the frontal heading direction. The minimum section encountered at an angle of 45° occurred alternately in the horizontal plane (dash-dotted green line) and the vertical plane (blue line), and the changes of speed occurred earlier than those recorded at an angle of 90°. (D) Larger of the two optic flow sums in the *horizontal* plane (dash-dotted green line) and the *vertical* plane (blue line), (mean(Max Σ*ω^45°^*) = 351.7±14.2°/s, the highest value peaks at Max(Max Σ*ω^45°^*) = 601°/s ), as well as the larger optic flow that would theoretically have been experienced at 45° at a constant speed (0.74 m/s), i.e., without the use of any speed control system by the bee (dashed black line, mean(Max Σ*ω^45°^_w/o_*_SpeedControl_) = 610.1±14°/s, the highest value peaks at Max(Max Σ*ω^45°^_w/o_*_SpeedControl_) = 1493°/s). The histograms on the right show the dispersion of the data. The faded colors around the curves give ± the standard error of the mean (s.e.m.).

In Figure 4B, the *larger mean* sums of the two lateral optic flows measured (Σω^90°^Lat = *ω^90°^_Rght _*+ *ω^90°^_Lft_*) and the two vertical optic flows measured at 90° (Σω^90°^Vert = *ω^90°^_Drsl_* + *ω^90°^_Vtrl_*) are plotted as a function of the abscissa *x*. In Figure 4C, the *larger mean* sums of the two lateral optic flows measured at 45° (Σω^45°^Lat = *ω^45°^_Rght_* + *ω^45°^_Lft_*) and the two vertical optic flows measured at 45° (Σω^45°^Vert = *ω^45°^_Drsl_* + *ω^45°^_Vtrl_*) are plotted versus the abscissa *x*.

To compare the variance of the larger Σω^90°^ with that of the larger Σω^45°^, the method and the extended *t-test* described by Zar were used ([37], Section 9.4, pp. 182–183). This involves taking the optic flow data normalized with respect to their respective means and making the following comparison based on the following one-tail hypotheses:

*H_0_*: variance Max(Σω^90°^) ≤ variance Max(Σω^45°^) **versus** *H_A_*: variance Max(Σω^90°^) > variance Max(Σω^45°^).

## Results

The top view (Figure 2A) and side view (Figure 2B) of the tunnel show the two successive tapered sections existing in the vertical and horizontal planes. A typical individual honeybee's trajectory recorded sideways through the insect netting and plotted every 100 ms is shown in Figure 2B. The honeybee's flight can be seen to have been quite *vertically centered* in the tunnel (mean height: 19±0.19 cm). It can be seen from Figure 2C that the honeybee gradually reduced its mean ground speed *Vx*_Bee_ down to the point where it approached the narrowest section of the tunnel, located 140 cm from the starting-point. The honeybee then increased its ground speed again as the tunnel widened out, first horizontally and then vertically. The mean trajectory and the mean ground speed of the 21 honeybees flying freely along the tapered tunnel are given in Figure 3B–3C, respectively. Figure 3A is a tentative diagram of the honeybees' trajectory in the horizontal plane, where the bees were assumed to take a laterally centered course, as suggested by previous experiments carried out in a narrow tapered tunnel [16]. Figure 3B gives the mean vertical trajectory of the honeybees plotted every 5 cm. The honeybees' mean course was clearly centered in the vertical plane of the tunnel (mean height *h* = 19±0.16 cm). Figure 3C gives the mean honeybees' ground speed as a function of the distance along the abscissa *x*. The honeybees clearly reduced their speed when approaching the narrowest section of the tunnel, and increased their speed again as the tunnel widened beyond this point (d.f. = 37, *F* = 28.2, *P*<0.001). A particular speed pattern emerged from the TukeyHSD post-hoc test on the ground speed profiles (Figure 3C, bottom trace). Up to point *x* = 60 cm on the abscissa, the speed was found to be constant (*NS*, *P*>0.05). Between *x* = 60 cm and *x* = 90 cm, the speed decreased significantly (*P*<0.001). Between *x* = 90 cm and *x* = 110 cm, the speed became constant again (*NS*, *P*>0.05). Between *x* = 95 cm and *x* = 130 cm, the speed decreased significantly once more (*P*<0.05) as the honeybees were about to reach the narrowest section of the tunnel. Lastly, from *x* = 130 cm to *x* = 180 cm, the speed gradually increased again (*P*<0.01) as the honeybees flew along the widening part of the tunnel. It is striking that the ground speed profile (Figure 3C, bottom trace) practically matched the minimum section profile recorded at 90° (Figure 1E).

To illustrate this point further, the minimum section profiles and the mean optic flows perceived both at 90° and at 45° by the honeybees are shown in parallel in Figure 4.

In Figure 4B, we plotted the *larger* of the two *mean optic flow sums* perceived by the bee at 90° (either laterally or vertically) (*ω^90°^_Rght_* + *ω^90°^_Lft_*, yellow line and *ω^90°^_Drsl_* + *ω^90°^_Vtrl_*, magenta line).

The *larger optic flow sum* first changed from lateral to vertical and from vertical to lateral just before reaching the points where the minimum section changed. The minimum section encountered at an angle of 90° narrowed twice along the tunnel, creating two constriction points:

1. The first narrowing occurred in the vertical plane, creating the first constriction point at *x* = 80 cm (Figure 4A, arrowhead n°1). As the bees approached this first constriction point, the *larger optic flow* sum increased. The minimum section remained steady between *x* = 80 cm to *x* = 110 cm, and the larger optic flow sum decreased, reaching a similar value to that perceived before the narrowing point (Max(Σ*ω* ^90°^)*_x_*_ = 60 cm_ = *609.5*±*29.6°/s* and Max(Σ*ω^90°^*)*_x_*_ = 100 cm_ *= 702.3*±*36.6°/s*).
2. The second narrowing occurred horizontally, creating the second constriction point (arrowhead n°2) at *x* = 140 cm. The larger of the two optic flow sums perceived increased until the honeybee reached the constriction. Then, as the tunnel widened, the larger optic flow sum gradually decreased again, reaching a similar value to that experienced before the narrowing point (Max(Σ*ω^90°^*)*_x_*_ = 110 cm_ = *624±28.8°/s* and Max(Σ*ω^90°^*)*_x_*_ = 170 cm_ *= 707.6*±*40.4°/s*).

One may wonder what these optic flow profiles would have looked like if the optic flow had not affected the bees' ground speed. In Figure 4B,C, the dash-dotted black lines show the dramatic change in the larger optic flow sum that the bee would have experienced at the viewing angles of 90° and 45° if it had kept flying at a *constant* ground speed (0.74 m/s), i.e. without the use of any speed control system.

The overall shape of the minimum section encountered at a viewing angle of 45° (Figure 4C) did not differ much from that encountered at a viewing angle of 90°. However, at 45°, the honeybee encountered each constriction in the frontal direction at a slightly shorter distance from the entrance than at 90°. Figure 4D shows the larger of the two optic flow sums generated at 45°: the overall shape of the larger optic flow sum profile observed at an angle of 45° was similar to that observed at 90°. The larger optic flow sum increased slightly as the tunnel narrowed and tended to reach a similar value to that recorded before the constriction point. In addition, the peaks in the larger optic flow sum profile were found to occur at practically the same places as the maximum optic flow perturbation induced by the narrowing sections. The first constriction was encountered at position x = 73 cm (Figure 4C, arrowhead n°3), whereas the larger optic flow sum (Max(Σ*ω^45°^*) ) occurred at x = 82 cm. The second constriction occurred at position x = 135 cm (arrowhead n°4), whereas the larger optic flow sum (Max(Σ*ω^45°^*)) occurred at x = 137 cm. Upon comparing the profiles shown in Figures 4B and 4D, the larger of the two optic flow sums generated (either vertically or laterally) was found to be better “stabilized” about a constant value at a viewing angle of 45° (mean(Max Σ*ω^45°^*) = 351.7±14.2°/s) than at a viewing angle of 90° (mean(Max Σ*ω^90°^*) = 711.8±24°/s). This conclusion was supported by comparisons between the variances of the larger optic flow sums obtained at 90° and at 45° (Max(Σ*ω^90°^*) and Max(Σ*ω^45°^*)): as shown by the histograms to the right of Figure 4B,D, the variance-to-the-mean ratio was distinctly lower at 45° than at 90° ( *t*_(36)_ = 2.99, p<0.01 ). If the honeybees' speed was not controlled, the honeybees would have perceived much larger maximum sum of the 2 opposite optic flows than what they actually perceived in our doubly tapered tunnel, which is shown by the dashed black lines in Figures 4B and 4D (mean(Max Σ*ω^90°^_w/o_*_SpeedControl_) = 1258.2±85°/s and mean(Max Σ*ω^45°^_w/o_*_SpeedControl_) = 610.1±14°/s ).

**Figure 5:**
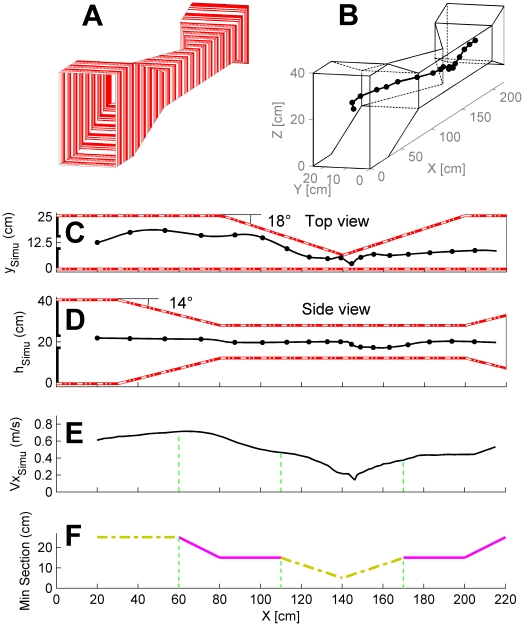
Simulated performances of the minimalist ALIS model in the same doubly-tapered tunnel. (A) Perspective view of the doubly-tapered tunnel lined with red and white stripes. Two tapered zones occur in this simulated tunnel: the first one is tapered in the vertical plane (from 30 cm to 80 cm, tapering angle 14°), and the second, in the horizontal plane (from 80 cm to 200 cm, tapering angle 18°). (B) Simulated bee's 3-D trajectory starting at initial coordinates x_0_ = 0.01 m; y_0_ = 0.135 m; z_0_ = 0.2 m, and at the speed *Vx*_oSIMU_ = 0.13 m/s. The time markers are plotted every 250 ms. (C) Trajectory in the vertical plane (x, z). The time markers are plotted every 250 ms. (D) Trajectory in the horizontal plane (x,y). The time markers are plotted every 250 ms. (E) Ground speed *Vx*_SIMU_ profile generated by the ALIS model based on two optic flow regulators: this profile accounts very satisfactorily for the minimum section of the doubly-tapered tunnel shown below. (F) Minimum section of the doubly-tapered tunnel along the abscissa. Due to the design of the tunnel, the minimum section was encountered alternately in the horizontal plane (dash-dotted yellow line) and the vertical plane (magenta line).

Figure 5 shows the flight path and the speed profile of a simulated agent equipped with the ALIS autopilot [22], flying along the same tunnel comprising two constrictions, in the vertical and horizontal planes. Figure 5C and 5D shows the trajectories in the horizontal plane (x, y) and the vertical plane (x,z), respectively. In the vertical plane, the simulated agent can be seen to fly roughly in the middle of the tunnel (this is because its “positioning optic flow set-point” is set at half of the “speed optic flow set-point”, see [38]). In the horizontal plane, however, the simulated agent followed one lateral wall. The large variations in the cross-sections occurring along the tunnel continuously disturbed the ALIS autopilot and did not give the simulated agent enough time to asymptotically reach the final horizontally and vertically centered position. The simulated agent nevertheless automatically kept a safe lateral clearance from the walls (Figure 5C) as well as a safe clearance from both the floor and the ceiling (Figure 5D), which brought it near the middle of the tunnel. The simulated agent can be seen to automatically slow down as the minimum cross-section of the tunnel narrows and to automatically accelerate again when the minimum cross-section widens (Figure 5E,F). Since the tunnel alternately narrows in the vertical and horizontal planes, the optic flow perceived laterally and vertically constraining the agents' speed alternately. The ALIS autopilot makes the simulated agent cross the doubly-tapered tunnel safely, in spite of major optic flow disturbances that alternately affect its eyes laterally, ventrally and dorsally. All in all, these results show that the ALIS-based simulated agent adopts a speed (Figure 5E) that is automatically adjusted to the minimum section profile (Figure 5F): the minimum section profile producing the largest optic flow.

## Discussion

In the experiments described here, honeybees were trained to fly along a specially designed tunnel comprising two successive tapering sections, the first of which was constricted in the vertical plane and the second, in the horizontal plane. During the trials, the honeybees, which entered the tunnel at half height, kept a centered position in the vertical plane along the whole the tunnel (Figure 2B, Figure 3B). The honeybees reduced their speed as the tunnel narrowed and speeded up again as the tunnel widened (Figure 2C, Figure 3C). The results of this experiment clearly show that the honeybees controlled their speed on the basis of all the surrounding optic flows (the left, right, ventral and dorsal optic flow). Two main points emerge from this study:

1. Honeybees react to a narrowing in the *vertical plane* by reducing speed in the same way as they do when they encounter a narrowing in the *horizontal plane* (Figure 2C, Figure 3C).
2. In reducing their speed, honeybees maintain the larger of the two perceived optic flow sums at a relatively constant value (Figure 4B–4D).

### Honeybees adjusted their speed in the same way, regardless of whether the tunnel narrowed vertically or horizontally

First, honeybees clearly reduced their speed when they encountered the first (vertical) tapering section of the corridor (Figure 3C). After training honeybees to fly along a corridor with horizontally tapered walls, Srinivasan et al. established that honeybees decreased their flight speed “*to hold the angular velocity of the image on the walls constant*” [16]. This previous finding provided definite evidence that the *lateral* optic flows are directly involved in honeybees' flight speed control system. The question still remained to be answered, however, as to whether the *ventral* optic flow is involved in the insects' flight speed control system. Previous studies on fruit flies [4], moths [39], and beetles [40] have shown that when following an odor plume, these insects flew faster when their distance from the floor increased. It was concluded that the insect may adjust its flight speed so as to maintain its ventral optic flow constant. In previous studies on honeybees [20], [21], various tunnels have been used, the floors of which were lined with stationary patterns of various kinds, such as 2-D patterns providing strong ventral optic flow cues, axial patterns providing weak ventral optic flow cues or a homogeneous pattern providing hardly any optic flow cues. The honeybees were found to fly on average at a lower height and a higher speed when only a few ventral optic flow cues were available.

In the present study, the honeybees were found to decrease their ground speed as they flew along the first vertically tapering part of the corridor, which perturbed both their dorsal and ventral optic flows (Figure 2, Figure 3).

In our doubly-tapered tunnel, the bee's ground speed *Vx_Bee_* showed small oscillations (Figure 2C) with a main frequency of 2.5±0.3 Hz on average, based on all the individual trajectories recorded. In a completely different condition (rotary drum condition), previous authors reported that a lateral peering occurred at a frequency of about 7 Hz [41]. In a straight, narrow tunnel, a lateral oscillation with a mean frequency of 4.7±1.6 Hz was reported to occur in various visual conditions ([42], pp. 51–52). This discrepancy between frequencies is probably attributable to the differences between experimental conditions. These oscillations in the bees' ground speed frequency might be partly due to the bee's visual speed control system being highly constrained and disturbed by our narrow doubly-tapered tunnel.

The performances of the bees shown in Figure 2,3 provide evidence that in addition to the *lateral* optic flows [16], [17] and the *ventral* optic flow [20], [21], the *dorsal* optic flow is involved in the speed control process. In the experiments presented here, it is noteworthy that the honeybees did not start to decrease their ground speed at the point where they encountered the vertical tapering (*x* = 30 cm), but rather at the point (*x* = 60 cm) where the local *vertical* section became narrower than the local *horizontal* section (Figure 1E, Figure 3C). Up to this point (*x* = 60 cm), the honeybees' speed depended on the local *horizontal* section, presumably since it was the smaller of the two sections. At *x* = 60 cm, the *vertical* section became narrower than the *horizontal* section: the larger optic flow sum of the two was therefore that involving the top and bottom walls, and this was the parameter used to determine the honeybee's speed. The opposite situation occurred later on (at *x* = 110 cm), when the local *horizontal* section became narrower than the local *vertical* section: it was therefore the *horizontal* section that took over as the parameter determining the bee's speed. The honeybees' ground speed therefore depended on the *minimum local cross-section* of the tunnel, regardless of whether this minimum value was reached on the *vertical* or *horizontal* plane.

### Maintaining the perceived optic flows at a constant level

The tapered tunnel greatly modified the optic flows throughout the visual field of the honeybees. The insects reacted to these disturbances by decreasing or increasing their speed accordingly. As the result of these changes of speed, the perceived optic flows were gradually restored to a similar level to that perceived by the insects before the disturbance. This can be seen from the fact that larger optic flow sum profiles were calculated at viewing angles of both 90° and 45° with respect to the tunnel x-axis (Figure 4B–4D). We therefore observed that the larger optic flow sum was stabilized (Figure 4B–4D) thanks to the decrease and the increase of the honeybees' speed.

In our doubly-tapered tunnel, the optic flow experienced by the bees was relatively high in comparison with that induced by other experimental conditions (bees' and wasps' learning flight [43]; bees flying along a *straight* tunnel [17]). However, the maximum sum of the optic flows experienced at a viewing angle of 90° by the bees in the doubly-tapered tunnel (maximum sum of either the vertical or the lateral optic flow sums: ∼710°/s was similar to the value obtained (∼650°/s) in another tapered tunnel by re-computing the data published in [16, figure 2]. In general, the optic flow values are relatively high in the case of bees flying along a tapered tunnel and in that of bees about to land [16]. In all these cases, bees actively change their speed when performing the task. In our doubly-tapered tunnel, the decreasing section brings the bee closer to either the walls, the floor or the ceiling. These major disturbances in the optic flows are then rejected by the bees' speed control system (figure 4B, figure 4D).

Various studies have shown that flying insects tend to hold the perceived optic flows constant, and that they do so by adjusting either their *distance* from the nearby surfaces (the floor or nearby walls) and/or their *ground speed* [4], [16], [18], [20], [39]. To explain the mechanism underlying this behavior, a control system called the *optic flow regulator* was developed, based on a feedback loop that consistently strives to maintain the perceived optic flow at a constant level [23], [12]. This control scheme - which relies exclusively on optic flow sensors and does not require any speed sensors or range sensors - was found to account for the height control abilities of several insect species flying in open spaces devoid of lateral textures [12], [15], [23], [44], [45].

The ALIS model we recently developed [22] extends the principle of the *optic flow regulator* [23], [38] to include the vertical dimension. The ALIS model is minimalistic, as it does not include the large optic flow receptive fields with which insects are endowed [10], [46]. The ALIS-based simulated trajectory (Figure 5) obtained in the same doubly-tapered tunnel to that used in the present experiments accounts quite well for the honeybees' vertical position and ground speed profiles observed (Figure 3B and 3C, respectively). It also accounts satisfactorily for the honeybees' performance in a high-roofed tunnel equipped with a moving floor [15]. Upon arriving above the moving part of floor (which moved in the same direction as the flying insect, thus reducing the ventral optic flow), the honeybee reacted by descending, while holding the same speed it had reached above the initial, stationary part of the floor. This finding can be explained by the fact that in the straight, high-roofed tunnel, the minimum cross-section (which was always the horizontal one) remained constant throughout the tunnel, hence yielding a constant groundspeed. In those conditions, the insect was left with decreasing its groundheight so as to retrieve the optic flow set point [15]. In the present study, where the tunnel tapered successively in the vertical and horizontal planes, the minimum cross-section alternated between the horizontal and vertical sections (Figure 1E). The honeybees' speed profile obtained (Figure 2C, 3C) may account for the fact that (i) the speed was no longer constrained to remain constant by a constant minimum cross-section, (ii) the steady vertical positioning (“vertical centering”: Figure 3B, 4B) revealed that the ground speed decreased so as to maintain the *larger of the two optic flow sums* (“left + right” optic flows or “ventral + dorsal” optic flows) constant whether the minimum cross-section was in the horizontal or vertical plane. These new experimental findings on flying bees are therefore fully consistent with the ALIS model [22], one outcome of which is that the groundspeed attained is proportional to the tunnel's smaller cross-section.

The ALIS dual optic flow regulator features two controllers (dynamic compensators) [22]: (i) a Proportional-Derivative (PD) controller in the *positioning feedback loop* (which is responsible for the *sway* and *heave* degrees of freedom), (ii) a Proportional-Integral (PI) controller in the *speed feedback loop* (which is responsible for the *surge* degree of freedom). But any kind of controller, including a simple proportional controller, would lead the simulated bee *to adjust the ground speed proportionally to the tunnel's smaller cross-section in a similar manner, as long as it ensures dynamic stability of the feedback loop*. *In other words, the nature of the controller does not affect the basis of the OF regulation scheme* [22], [23].

The optic flow sensors used in the present simulation (Figure 5) were based on a previously described fly-inspired “time of travel scheme” ([47], [48] (see also further details in [23]). Since the optic flow sensor was implemented here to operate inside a feedback loop (the optic flow regulator) about an angular velocity constant value (the optic flow set point), there is a sole requirement with respect to the optic flow sensor: its characteristic has to be a monotonic function of the angular velocity in the range about the optic flow set-point. Various optic flow sensor schemes give a monotonic characteristic curve, including that of correlation-type motion detectors [49], [50], at least in a given range [51]. In other words, the performances resulting from the use of optic flow regulators – which are the basis of the ALIS model –, do not depend on how the optic flow is assessed.

Analysis of the *larger of the two optic flow sums* showed that their variance was lower at an angle of 45° than at 90°. This suggests that optic flow information originating from frontal regions of the visual field contributes to improving the insects' flight performances, as established by Baird et al. (2010) [19]. To make even better use of the present findings, it is proposed in the future to develop a more sophisticated ALIS model, in which the optic flows occurring in larger fields of view, including frontal optic flows, will be regulated and therefore kept constant. Frontal optic flow information has been previously used in robot design to solve obstacle avoidance problems [47], [48], [52], ground avoidance problems [35], [36], [44], [45], and speed control problems [53], [54].

As far as insects' flight control is concerned, the *optic flow regulator* concept has several advantages. It makes an insect automatically select both a safe speed and a safe position in the surrounding environment without any need for onboard ground speed sensors or range sensors whatsoever. The only sensors required are optic flow sensors, the output signal of which grows with the ground speed-to-ground height ratio. This control system also provides an interesting, robust and inexpensive means of piloting an aircraft or a spacecraft, as long as there are sufficiently large numbers of photons and contrasting features in the environment [55], [56].

Optic flow processing and visuomotor control systems in insects can be expected to match the natural motion signals triggered by flight in specific environments [57]. Sensitivity to the dorsal optic flow can be said to meet ecological constraints. It enables flying honeybees to keep a safe speed while crossing complex foraging environments, where dorsally located objects abound and have to be sensed just as much as ventrally or laterally located objects. This is the case in particular whenever bees inspect dense patches of vegetation, flying under the foliage and flowers in search of nectar.

The cartoon-like tunnel experiments described here need to be extended to free 3-D space, real-life conditions and variously structured environments. Further studies are also required to test the relevance of our model in more natural environments and improve our understanding of insects' flight control systems.

## Supporting Information

Figure S1 **A honeybee flying along the doubly-tapered tunnel.** The photograph was taken at the entrance of the tunnel.
(TIF)

Movie S1 **An animated 3D view of the doubly-tapered tunnel.**
(MP4)
