# Supplementary figures and images for: Honeybees' Speed Depends on Dorsal as Well as Lateral, Ventral and Frontal Optic Flows
Source: PLoS One. 2011 May 12;6(5):e19486. doi: 10.1371/journal.pone.0019486 (PMC3093387; doi:10.1371/journal.pone.0019486)

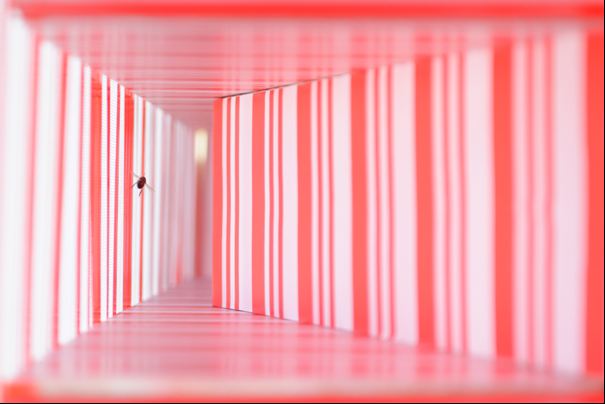

Supplement: Figure S1 — A honeybee flying along the doubly-tapered tunnel. The photograph was taken at the entrance of the tunnel. (TIF) [file pone.0019486.s001.tif]
